# Androgen Inhibits Abdominal Fat Accumulation and Negatively Regulates the *PCK1* Gene in Male Chickens

**DOI:** 10.1371/journal.pone.0059636

**Published:** 2013-03-27

**Authors:** Jinlin Duan, Fan Shao, Yonggang Shao, Junying Li, Yao Ling, Kedao Teng, Hongwei Li, Changxin Wu

**Affiliations:** 1 College of Animal Science and Technology, China Agricultural University, Beijing, China; 2 College of Animal Science, Xinjiang Agricultural University, Urumqi, Xinjiang, China; 3 College of Veterinary Medicine, China Agricultural University, Beijing, China; Hospital Infantil Universitario Niño Jesús, CIBEROBN, Spain

## Abstract

Capons are male chickens whose testes have been surgically incised. Capons show a significant increase in fat accumulation compared to intact male chickens. However, while caponization leads to a significant reduction in androgen levels in roosters, little is known about the molecular mechanisms through which androgen status affects lipogenesis in avian species. Therefore, investigation of the influence of androgens on fat accumulation in the chicken will provide insights into this process. In this study, Affymetrix microarray technology was used to analyze the gene expression profiles of livers from capons and intact male chickens because the liver is the major site of lipogenesis in avian species. Through gene ontology, we found that genes involved in hepatic lipogenic biosynthesis were the most highly enriched. Interestingly, among the upregulated genes, the cytosolic form of the phosphoenolpyruvate carboxykinase (*PCK1*) gene showed the greatest fold change. Additionally, in conjunction with quantitative real-time PCR data, our results suggested that androgen status negatively regulated the *PCK1* gene in male chickens.

## Introduction

Capons are male chickens whose testes have been surgically incised. Caponization can produce a unique type of poultry meat grown for a specialized market because the capon meat is more tender, juicy, and flavorful than that of intact cockerels [1]. A possible reason for this difference in meat quality is that caponization results in a greater increase in subcutaneous, intercellular, and abdominal fat compared with fat accumulation in intact cockerels [2]–[5]; this increase in fat improves meat flavor.

Caponization has been shown to lead to a significant reduction in androgen levels in roosters. Androgen is a steroid hormone, and the primary and most well-known androgen is testosterone; other less-important androgens include dihydrotestosterone and androstenedione. In male mammals, androgen status plays an important role in adipogenesis [6]–[12]; however, little is known about the molecular mechanisms through which androgen status affects lipogenesis in avian species. Therefore, investigation of the influence of androgens on fat accumulation in the chicken will provide interesting insights into this process.

The liver is the main organ of lipogenesis in poultry [13]. In a previous study, Chen et al [14] suggested that an increase in the activity and mRNA levels of malic enzyme 1 (ME1) may be positively associated with hepatic lipogenesis in male White Leghorn chickens. However, the global expression profiles of related hepatic lipogenic genes following caponization have not been elucidated.

Microarray technology is a powerful method for profiling gene expression patterns of thousands of genes in a single experiment, and is therefore widely applied to identify the tissue- and disease-specific conditions under which genes are expressed [15]–[20]. In many respects, the 2 types of labeled targets, i.e., cRNA and cDNA, are considered to be equivalent for microarray analysis. However, cRNA has proven to be advantageous for experiments with small amounts of starting RNA [21], [22] and is required for Affymetrix microarray analysis. In this study, Affymetrix microarray technology was employed to analyze the gene expression profiles of capons and intact male chickens. We performed gene expression profiling using livers from these 2 types of roosters and explored the possible molecular mechanisms governing lipid accumulation after caponization. From our results, we suggest that the gene encoding phosphoenolpyruvate carboxykinase 1 (PCK1) plays an important role in fat accumulation and is negatively regulated by androgen status in male chickens.

## Materials and Methods

### Animals

Healthy male single-comb White Leghorn chickens were selected from the Experimental Poultry Genetic Resource and Breeding Chicken Farm of China Agricultural University. All chickens were housed in a modern, nationally certified animal facility under the supervision of board-certified veterinarians. This study was carried out in strict accordance with the Regulations for the Administration of Affairs Concerning Experimental Animals of the State Council of the People’s Republic of China. The protocol was approved by the Committee on Experimental Animal Management of China Agricultural University.

### Experimental Design

Sixty male chickens of the same age (ages 9 or 17 weeks) and with similar body weights were randomly divided into 3 groups (20 individuals per group per age). One group was taken as the control and the other 2 groups were caponized.

The caponization procedure was performed according to previously described methods [2], [5], [23]. Before the surgical operation, male chickens were prohibited from feeding for 12 h. The incision site was sterilized with veterinary external disinfectants. A 2–3-cm lateral incision was made at the second or third last rib, and the chicken’s 2 testes were removed. Veterinary external disinfectant was applied again to the incision site, which was closed with surgical sutures. After caponization, there was a 4-week recovery, followed by a 10-week feeding trial.

After caponization, 1 of the 2 caponized groups was used for implanting testosterone because the removal of testes leads to a greater decrease in body testosterone content. Therefore, implantation of testosterone would allow us to investigate the effects changes in body androgen status on fat biosynthesis. The testosterone implantation procedure was performed according to previously reported methods [2], [5], with modifications, and the testosterone amount implanted was based on a previously described method [5]. Exogenous testosterone was purchased from Sigma (USA) and formed into pellets (every pellet contained 18 mg testosterone). Using implantation guns from the Animal Reproduction Laboratory of China Agricultural University (Beijing), testosterone was implanted subcutaneously at the back of the chicken’s neck during the 10-week feeding trial (18 mg per individual dose with a total of 3 doses over 10 weeks; the “embed group”). The other 2 groups were called the control group (including only intact male chickens) and the capon group (including only caponized male chickens who were not given testosterone implants), respectively. All chickens were sacrificed by qualified technicians in a clean slaughterhouse by having their carotid arteries severed with clean neck cutters under anesthesia.

### Determination of Abdominal Fat Content and Serum Hormone Concentrations

Blood samples were taken from the brachial veins of chickens following 12-h fasting from food and water prior to slaughter and were then stored in anticoagulant blood vessels at 4°C until use. Determination of sex hormone content using these blood samples was completed at the Sino-UK Institute of Biological Technology (Beijing). Serum testosterone and estradiol concentrations were determined by a previously described method [24] using a γ-counter (GC-911) with a radioimmunoassay kit.

After slaughter, the abdominal fat and liver tissues were removed immediately. Abdominal fat was weighed, and liver tissues were frozen in liquid nitrogen and stored at −80°C for further analysis.

### Liver Microarray Analysis

We randomly selected 9 individuals from the control chickens and capons at 23 weeks of age. After slaughter, total RNA was isolated from liver tissues using Trizol (Invitrogen, Paisley, UK). We used a pooled design in order to obtain a sufficient amount of RNA to run an array. Nine RNA samples from control chickens or capons were randomly divided into 3 pools to give 3 RNA samples per pool. RNA integrity was electrophoretically verified by ethidium bromide staining and by OD_260_/OD_280_ nm absorption ratio (>1.95). Next, we prepared the cRNA and microarray chips following the technical manual for GeneChip expression analysis provided by Affymetrix (File S2). All liver microarray analyses were performed at the Bioassay Laboratory of CapitalBio Corporation (18 Life Science Parkway, Changping District, Beijing, China; http://www.capitalbio.com).

First- and second-strand cDNA were synthesized from total RNA (∼1 µg) using the SuperScript II system (Invitrogen, CA, USA). After a clean up and quality check of the double-stranded cDNA, an in vitro transcription reaction was conducted with the Enzo RNA Transcript Labeling Kit (Affymetrix, Santa Clara, CA) to produce biotin-labeled cRNA from the cDNA. The cRNA was then purified with the RNeasy Mini Kit (Qiagen, Valencia, CA) and fragmented for hybridization analysis. Finally, the fragmented cRNA was hybridized with the Chicken Liver Microarray (Affymetrix) in a hybridization cocktail. Hybridization took place overnight (16 h) at 45°C in a GeneChip Hybridization Oven 640 at 60 rpm (Affymetrix), followed by washing and staining with streptavidin-phycoery-thrin (SAPE, Molecular Probes, Eugene, OR) as described in the Affymetrix GeneChip Expression Analysis Technical Manual (File S2). The distribution of fluorescent material on the array was imaged using the GeneArray Scanner 3000 (Affymetrix). Microarray Suite (MAS) Version 5.0 and GeneChip Operating Software (GCOS), supplied by Affymetrix, were used for gene expression analysis. High-density oligonucleotide array probe level data were normalized using previously described methods [25].

Significance analysis of microarrays (SAM) is a method that can be used to identify differentially expressed genes. Each gene was assigned a score on the basis of its change in gene expression relative to the standard deviation of repeated measurements. Genes with scores that are significantly higher than the expected score were termed differentially expressed. The expected score was calculated by permuting the measurements and then taking the average score for all the permuted scores as the expected score. To control the type I error rate for multiple-hypothesis testing, SAM was used to fix the rejection region and then estimate its corresponding error rate. SAM applies the methodology to both the positive false discovery rate and q-value as presented in previous studies [26]. To identify significantly differentially expressed genes, we used the following criteria: fold change, ≥2 or ≤0.5; q-value, ≤5%. Gene ontology analysis was conducted at http://www.geneontology.org, and the pathway analysis was performed by KEGG (http://www.genome.jp/kegg).

### Quantitative Real-time PCR (qRT-PCR)

Twelve chicken livers were randomly selected from each of the 3 groups. Total RNA was extracted from the livers. cDNA was synthesized from 1 µg total RNA with M-MLV Reverse Transcriptase (Promega). Aliquots of cDNA were used as a template for real-time PCR. Reactions contained primers and probes for *PCK1* or *ME1* genes or primers and a probe for glyceraldehyde 3-phosphate dehydrogenase (*GAPDH*), which served as a reference gene. Each reaction contained cDNA derived from 20 ng total RNA. Three replicates were performed for each reaction.

qRT-PCR was carried out using a CFX Connect Real-Time PCR Detection System (Bio-R, Hercules, California, USA) in a Bio-Rad Real Time-PCR9600. Relative expression of target genes was calculated by a previously described method [27]. First, 12 livers from each of the control and capon groups were selected for carrying out qRT-PCR. Then, if significant differences were found between the 2 groups, irrespective of age, 12 livers from the embed group were selected for qRT-PCR as well.

Primer sets for PCK1 (forward, 5′-GCAGGGGTTATGATGAGAAGT-3′; and reverse, 5′-ACGGATCACAGTTTTGAAGAC-3′), ME1 (forward, 5′-CTGGAGTTGCTCTTGGTGT-3′; and reverse, 5′-TCCTGTAGGCTTCTTCTGC-3′), and the housekeeping internal control gene GAPDH (forward, 5′-GAAACCAGCCAAGTATGATG-3′; and reverse, 5′-ACCATTGAAGTCACAGGAGA-3′) were designed based on the sequences published in GenBank and using Primer Premier 5.0 software.

### Statistical Analysis

All statistical analyses were performed with the GLM procedure in SAS 9.1 software (SAS Institute, 1990). Tests of differences were carried out using Duncan’s new multiple range method [28] and values are presented as the mean ± standard error (SE).

## Results

### Abdominal Fat Content and Serum Sex Hormone Content

In previous observations, abdominal fat content and blood sex hormone content were shown to exhibit significant differences in capons compared with intact male chickens [2]–[5]. We therefore compared the 2 indexes among the different groups. As shown in Table 1, in White Leghorn male chickens aged 23 or 31 weeks, the capon group exhibited a greater increase in abdominal fat content than the embed and control groups, and there was no significant difference in abdominal fat between the embed and control groups (*P*>0.05). This result indicated that caponization enhanced abdominal fat deposition and that implantation of testosterone significantly inhibited abdominal fat deposition.

**Table 1:** Abdominal fat content of White Leghorn male chickens at different ages for the 3 groups.

Group	Abdominal fat content (g)	
	23 weeks of age*	31 weeks of age*
Capon	10.55±1.53^β^ (n = 20)	4.25±0.78^bβ^ (n = 20)
Embed	0.00±0.00^α^ (n = 20)	1.44±0.75^a^ (n = 20)
Control	0.00±0.00^α^ (n = 20)	0.00±0.00^α^ (n = 20)

Note: All values are depicted as means ± SE.

α,β *P*<0.01;

a,b *P*<0.05;

* age at the end of the experiment.

**Figure 1:**
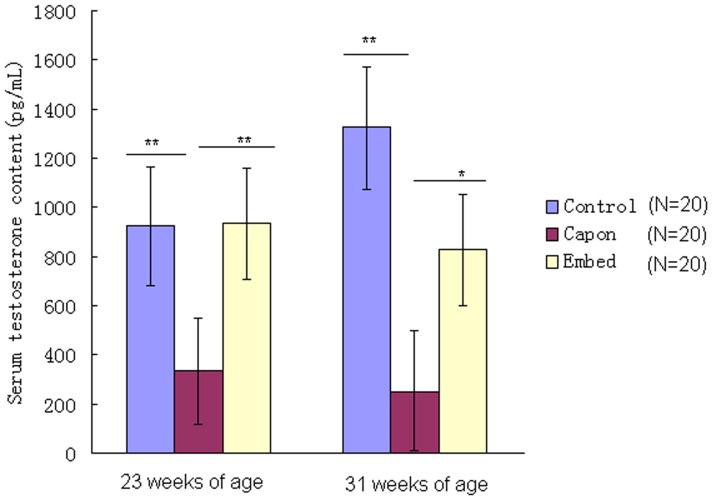
Serum testosterone content in the different groups. Serum testosterone content was determined in chickens from the control, capon and embed groups. *, *P*<0.05; **, *P*<0.01.

**Figure 2:**
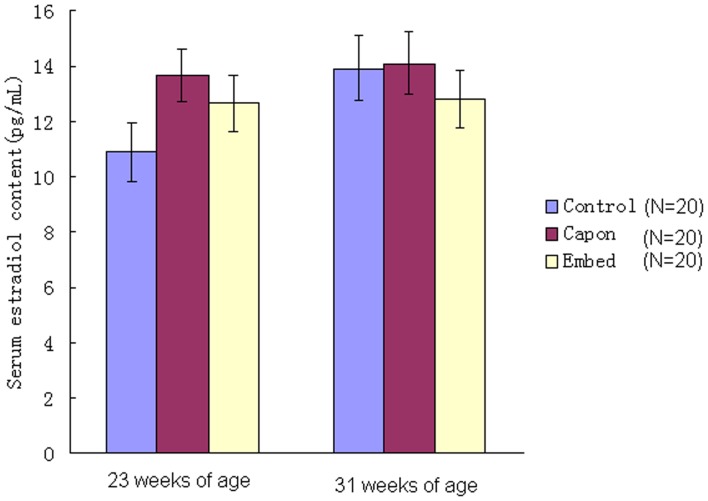
Serum estradiol content in the different groups. Serum estradiol content was determined in chickens from the control, capon and embed groups. *, *P*<0.05; **, *P*<0.01.

As shown in Figure 1, after caponization, serum testosterone content decreased dramatically in the capon group compared with the control and embed groups, irrespective of age, but showed no significant difference between the control and embed groups (*P*>0.05). The results also indicated that implantation of testosterone resulted in a significant recovery in serum testosterone content in capons. In contrast, serum estradiol levels did not differ among all groups, irrespective of age (*P*>0.05; Figure 2).

### Liver Microarray Analysis

Affymetrix microarray technology was used to analyze the gene expression profiles of chicken livers from the capon and control groups. Of the 38536 probes analyzed (File S3), 79 genes were upregulated by at least 2-fold, and 42 genes were downregulated by at least 2-fold in the livers of capons (File S1, Table 2). Gene ontology enrichment analysis indicated that the largest proportion of upregulated genes was involved in metabolic pathways, whereas genes involved in lipid metabolism were the most highly enriched (Table 3). Furthermore, pathway analysis by KEGG showed that genes with differential expression were mainly involved in lipid metabolism and Jak-STAT signaling pathways (Table 3).

**Table 2:** Genes differentially expressed (q <0.05) in capons’ livers compared to control livers of male White Leghorn chickens.

	Gene symbol	Gene or function	q-value	FC
**Upregulated**1	PCK1	phosphoenolpyruvate carboxykinase 1 (soluble)	0	28.87
2	ABCB1	ATP-binding cassette, sub-family B (MDR/TAP), member 1	0	6.26
3	MOGAT1	monoacylglycerol O-acyltransferase 1	0	5.80
4	CDKN2B	cyclin-dependent kinase inhibitor 2B (melanoma, p16, inhibits CDK4)	0.008	5.48
5	FABP1	fatty acid binding protein 1, liver	0	4.94
6	ABCG5	ATP-binding cassette, sub-family G (WHITE), member 5 (sterolin 1)	0	4.61
7	RBM38	RNA binding motif protein 38	0	4.29
8	LOC428660	similar to very large inducible GTPase-1	0	3.90
9	CHAC1	ChaC, cation transport regulator homolog 1 (*E. coli*)	0.005	3.65
10	GCLC	glutamate-cysteine ligase, catalytic subunit	0.009	3.65
11	RCJMB04_16d24	ELOVL family member 6, elongation of long chain fatty acids	0	3.37
12	GALE	UDP-galactose-4-epimerase	0	3.36
13	NCAM1	Neural cell adhesion molecule 1	0	3.30
14	APOA4	apolipoprotein A-IV	0	3.27
15	ADFP	Adipose differentiation-related protein	0.010	3.24
16	SIK1	salt-inducible kinase 1	0.003	3.22
17	TMCC2	transmembrane and coiled-coil domain family 2	0	3.16
18	BRP44L	brain protein 44-like	0.005	3.13
19	ELOVL2	elongation of very long chain fatty acids-like 2	0	3.12
20	ALDH18A1	aldehyde dehydrogenase 18 family, member A1	0.006	2.99
21	RCJMB04_5k4	selenoprotein I	0	2.93
22	DBC1	deleted in bladder cancer 1	0.019	2.91
23	ELOVL1	elongation of very long chain fatty acids	0.007	2.87
24	LOC420707	hypothetical gene supported by CR386894	0	2.73
25	WDR66	WD repeat domain 66	0.011	2.73
26	SEC22C	SEC22 vesicle trafficking protein homolog C (*S. cerevisiae*)	0	2.70
27	ABI3	ABI gene family, member 3	0	2.68
28	EREG	epiregulin	0.026	2.68
29	MFSD2	major facilitator superfamily domain containing 2	0	2.66
30	FICD	FIC domain containing	0.014	2.64
31	C7orf23	chromosome 7 open reading frame 23	0.008	2.61
32	FADS2	Fatty acid desaturase 2	0.003	2.60
33	SERPINA3	serpin peptidase inhibitor, clade A, member 3	0.019	2.58
34	SPG20	spastic paraplegia 20 (Troyer syndrome)	0.004	2.56
35	IL10RB	interleukin 10 receptor, beta	0.005	2.55
36	SLC39A8	solute carrier family 39 (zinc transporter), member 8	0.005	2.54
37	SH3YL1	SH3 domain containing, Ysc84-like 1 (*S. cerevisiae*)	0	2.53
38	CRAT	carnitine acetyltransferase	0.031	2.52
39	SEBOX	SEBOX homeobox	0.012	2.49
40	SOCS3	suppressor of cytokine signaling 3	0.019	2.44
41	LOC421910	similar to Acp1 protein	0.004	2.40
42	SOCS1	suppressor of cytokine signaling 1	0.006	2.35
43	ATOH8	Atonal homolog 8 (*Drosophila*)	0.006	2.29
44	IRG1	immunoresponsive 1 homolog (*M. musculus*)	0.020	2.28
45	ECE1	endothelin converting enzyme 1	0	2.25
46	FADS1	fatty acid desaturase 1	0.005	2.25
47	ABCD3	ATP-binding cassette, sub-family D (ALD), member 3	0.005	2.23
48	SLC16A5	solute carrier family 16, member 5 (monocarboxylic acid transporter 6)	0	2.22
49	THRSP	thyroid hormone responsive (SPOT14 homolog, *R. norvegicus*)	0	2.22
50	GNPNAT1	glucosamine-phosphate N-acetyltransferase 1	0	2.19
51	LOC768655	similar to pim-3 protein	0	2.19
52	PHOSPHO1	phosphatase, orphan 1	0.004	2.19
53	RCJMB04_28i8	Fas (TNFRSF6) binding factor 1	0.011	2.19
54	SLC41A1	Solute carrier family 41, member 1	0.015	2.19
55	LOC418109	hypothetical LOC418109	0.016	2.18
56	ME1	malic enzyme 1, NADP(+)-dependent, cytosolic	0.013	2.18
57	ZYG11B	zyg-11 homolog B (*C. elegans*)	0	2.18
58	C4orf33	Chromosome 4 open reading frame 33	0	2.16
59	CYP4B1	cytochrome P450, family 4, subfamily B, polypeptide 1	0	2.16
60	LOC416033	similar to MGC80162 protein	0	2.16
61	YARS	tyrosyl-tRNA synthetase	0.013	2.15
62	CPT1A	carnitine palmitoyltransferase 1A (liver)	0	2.11
63	GATA5	GATA binding protein 5	0.003	2.10
64	SLC16A1	solute carrier family 16, member 1 (monocarboxylic acid transporter 1)	0.004	2.10
65	ACAA1	acetyl-CoenzymeA acyltransferase 1	0.003	2.09
66	CCDC53	Coiled-coil domain containing 53	0.003	2.09
67	HRASLS	HRAS-like suppressor	0.009	2.09
68	FABP7	fatty acid binding protein 7, brain	0	2.08
69	LOC422046	similar to LOC494798 protein	0.005	2.08
70	CCDC13	coiled-coil domain containing 13	0.004	2.05
71	ICER	ICER protein	0.012	2.05
72	UBE2J1	ubiquitin-conjugating enzyme E2, J1 (UBC6 homolog, *S. cerevisiae*)	0	2.05
73	ABHD3	abhydrolase domain containing 3	0.003	2.04
74	NXN	nucleoredoxin	0.012	2.04
75	CYCS	cytochrome c, somatic	0.005	2.03
76	IL20RA	interleukin 20 receptor, alpha	0.004	2.01
77	SERPINA12	serpin peptidase inhibitor, clade A, member 12	0.014	2.01
78	USP4	ubiquitin specific peptidase 4 (proto-oncogene)	0	2.01
79	AGPAT4	1-acylglycerol-3-phosphate O-acyltransferase 4	0	2.00
**Downregulated**				
1	PER3	period homolog 3 (*Drosophila*)	0.008	−2.01
2	SLC16A10	Solute carrier family 16, member 10 (aromatic amino acid transporter)	0.008	−2.01
3	ARHGAP24	Rho GTPase activating protein 24	0	−2.02
4	N4BP2L1	NEDD4 binding protein 2-like 1	0.005	−2.02
5	PTPRG	protein tyrosine phosphatase, receptor type, G	0.005	−2.04
6	CYP3A80	cytochrome P450 3A80	0.008	−2.07
7	HAL	histidine ammonia-lyase	0.022	−2.08
8	RCJMB04_34j1	protein kinase-like protein SgK196	0.007	−2.08
9	AKR1D1	aldo-keto reductase family 1, member D1	0.038	−2.09
10	LOC416335	apical protein 2	0.005	−2.12
11	FOXP2	forkhead box P2	0.019	−2.13
12	SLC2A5	solute carrier family 2, member 5	0.026	−2.13
13	KYNU	kynureninase (L-kynurenine hydrolase)	0.018	−2.14
14	ZBTB16	zinc finger and BTB domain containing 16	0.011	−2.18
15	SLC7A2	solute carrier family 7, member 2	0.015	−2.19
16	TC2N	tandem C2 domains, nuclear	0.048	−2.22
17	COL6A3	collagen, type VI, alpha 3	0.027	−2.23
18	SRGAP1	SLIT-ROBO Rho GTPase activating protein 1	0	−2.29
19	BUB1	BUB1 budding uninhibited by benzimidazoles 1 homolog (*S. cerevisiae*)	0.006	−2.32
20	PTPN1	protein tyrosine phosphatase, non-receptor type 1	0	−2.40
21	GAL13	beta-defensin 13	0.014	−2.62
22	LOC426431///OAT	hypothetical LOC426431///ornithine aminotransferase	0.014	−2.65
23	FABP5	fatty acid binding protein 5	0	−2.69
24	ASAH2	N-acylsphingosine amidohydrolase 2	0.042	−2.71
25	PPAT	phosphoribosyl pyrophosphate amidotransferase	0.013	−2.71
26	LPIN1	lipin 1	0.021	−2.76
27	HNF4A	hepatocyte nuclear factor 4, alpha	0.013	−2.84
28	CYP1A4	cytochrome P450 1A4	0.009	−2.89
29	IL1RL1	interleukin 1 receptor-like 1	0.008	−3.01
30	LOC769659///OAT	ornithine amino transferase	0.009	−3.18
31	CYP7A1	cytochrome P450, family 7, subfamily A, polypeptide 1	0.009	−3.20
32	LOC421091	similar to transthyretin	0.019	−3.25
33	SOCS2	suppressor of cytokine signaling 2	0	−3.25
34	GLDC	glycine dehydrogenase (decarboxylating)	0	−3.28
35	FKBP5	FK506 binding protein 5	0.008	−3.44
36	EPAS1	endothelial PAS domain protein 1	0	−3.46
37	CHIA	chitinase, acidic	0	−3.88
38	GPT2	glutamic pyruvate transaminase (alanine aminotransferase) 2	0	−3.91
39	IGSF21	immunoglobin superfamily, member 21	0	−4.64
40	UPP2	uridine phosphorylase 2	0.005	−6.37
41	LOC770705	Similar to pol	0.016	−6.93
42	CHIA///LOC768786	chitinase, acidic///similar to CBPch04	0	−7.12

**Note:** Accession numbers of the genes are shown in File S1.

**Table 3:** Gene categories according to pathway.

Pathway	*P*-value	Genes^a^
Biosynthesis of unsaturated fatty acids	1.50E−09	**ACAA1**; **RCJMB04_16d24;** **ELOVL2; FADS1;** **FADS2**
Bile acid biosynthesis	2.24E−05	**ACAA1;** AKR1D1; CYP7A1
Jak-STAT signaling pathway	2.26E−05	**IL10RB;**
ABC transporters - General	8.78E−05	**ABCD3;**
Fatty acid metabolism	1.08E−04	**CYP4B1;**
Glutamate metabolism	0.0015	PPAT;
Aminosugars metabolism	0.0017	CHIA;
Alanine and aspartate metabolism	0.0026	**CRAT;**
Retinol metabolism	0.0029	**CYP4B1;**
Drug metabolism - other enzymes	0.0033	UPP2;
Pyruvate metabolism	0.0043	**ME1;**
Ubiquitin mediated proteolysis	0.0051	**SOCS3;**
Glycerophospholipid metabolism	0.0092	**PHOSPHO1;**

a Genes in bold are upregulated.

### Expression Patterns of the PCK1 and ME1 Genes

**Figure 3:**
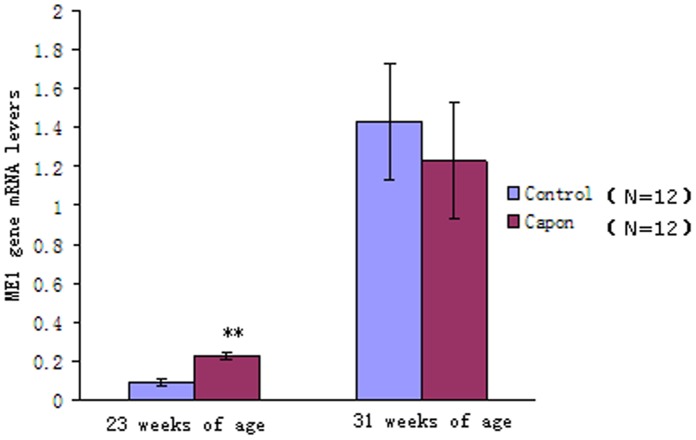
Relative mRNA expression of the *ME1* gene in the different groups. Twelve livers each from the control and capon groups (of chickens with different ages) were selected for carrying out qRT-PCR. Results are presented as means ± SE; *, *P*<0.05; **, *P*<0.01.

Among the upregulated genes, the largest fold change was observed in the cytosolic form of *PCK1*, while only a 2-fold change was found in *ME1* (Table 2). In a previous study, the *ME1* gene was suggested to play a key role in fat biosynthesis in capons [14]. At the same time, microarray analysis showed that the *PCK1* gene exhibited the largest fold increase in capons. Therefore, qRT-PCR was carried out to analyze the expression patterns of the *ME1* and *PCK1* genes. Interestingly, we found that the expression of the *ME1* gene differed significantly between the capon and control groups only at 23 weeks of age, but not at 31 weeks of age (Figure 3). Thus, our results showed that expression of the *ME1* gene was associated with caponization age but not androgen status in male chickens.

**Figure 4:**
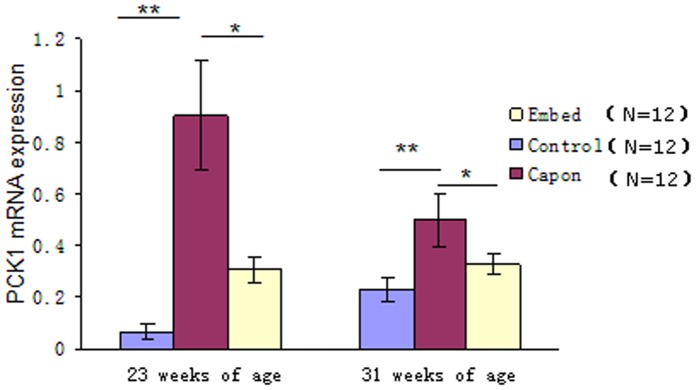
Relative mRNA expression of *PCK1* in different groups. Twelve livers each from the control and capon groups (at different ages) were selected for carrying out qRT-PCR. When significant differences were found between the 2 groups, 12 livers from the embed group were also subjected to qRT-PCR. Results are presented as means ± SE; *, *P*<0.05; **, *P*<0.01.

In contrast, there was a significant difference in the expression of the *PCK1* gene between the control and capon groups and between the capon and embed groups, irrespective of age. At the same time, the expression of the *PCK1* gene showed no significant difference between the control and embed groups, irrespective of age (Figure 4). These results indicated that the expression of the *PCK1* gene was negatively regulated by androgen status.

## Discussion

### Androgen Status Negatively Affected Fat Accumulation in Male Chickens

Castration has been reported to result in an increase in fat accumulation over the longissimus muscle in male bulls or rams, more backfat in male boars [29], and an obvious increase in subcutaneous, intercellular, and abdominal fat in male chickens [2]–[5], which is consistent with the findings of our present study. Additionally, an increase in proliferation capacity and a loss of differentiation capacity were observed in epididymal pre-adipocytes in castrated rats [7].

Castration primarily decreases androgen levels in male animals due to the removal of the male testes. The primary and most well-known androgen is testosterone, which is an important determinant of body composition in male mammals [30], [31]. In men, abdominal obesity is usually associated with low serum testosterone levels [32]–[34]. At the same time, testosterone supplementation in healthy, young, hypogonadal men can result in a decrease in fat mass [35]–[39]. Likewise, testosterone supplementation increases skeletal muscle mass and decreases fat mass in mice [11]. In our study, we also found that serum testosterone levels were negatively correlated with abdominal fat accumulation in male chickens, irrespective of age while testosterone implantation resulted in a significant decrease in abdominal fat, which is consistent with a previous observation [14]. The results suggested that androgen status negatively affected fat accumulation in male chickens.

### Liver Microarray Analysis

Capons can accumulate lipids in the body, which enhances flavor and meat juiciness when compared with intact cockerels [1], [5], [40]. Therefore, it was expected that the largest proportion of upregulated genes would be involved in lipid metabolism. Additionally, previous studies have indicated that the Jak-STAT signaling pathway plays a key role in innate immunity [41]–[43]. Thus, our results suggested that caponization increases the immune response in male chickens, which is consistent with another previous study [44].

### Androgen Status Negatively Influenced PCK1 Gene Expression in Male Chickens

PCK1 catalyzes the conversion of oxaloacetate to phosphoenolpyruvate, the rate-limiting step in hepatic and renal gluconeogenesis and adipose glyceroneogenesis, and is expressed at high levels in liver, kidney, and adipose tissue [45]. In the liver, expression of the *PCK1* gene at the transcriptional level is stimulated by a number of hormones, including glucagon, cAMP, glucocorticoids, and thyroid hormone [46]–[50], but is inhibited by insulin [48], [51], [52]. However, the mechanism through which testosterone regulates expression of the *PCK1* gene has not been reported. Importantly, the results of our current study suggested that testosterone negatively regulates *PCK1* mRNA.

An increasing number of studies have shown that the *PCK1* gene plays a crucial role in multiple physiological processes in mammalian species and is involved in obesity, insulin resistance (type 2 diabetes mellitus, T2DM), and the mammary gland [53]–[65]. In chicken livers, the main form of PCK is mitochondrial PCK, also called PCK2; in contrast to PCK2, PCK1 plays an important role in gluconeogenesis in the kidney [66], [67]. Presently, the *PCK1* gene has not been reported to be involved in glyceroneogenesis in avian livers. In our study, the results showed that *PCK1* mRNA expression had a positive relationship with abdominal fat accumulation in male chickens, suggesting that the *PCK1* gene plays a crucial role in lipogenesis in capons.

### The Mechanism through which Androgen Status Affects Adipogenesis in Male Chickens

In castrated rats, androgen status is thought to affect adipogenesis from deep intra-abdominal pre-adipocytes through altered MAP kinase cascade/Fos signaling pathways [8]. In men, testosterone affects adipogenesis by regulation of the activities of lipoprotein lipase (LPL) and hormone-sensitive lipase (HSL) [12], [68]. A previous study indicated that caponization increased *ME1* mRNA expression at 26 weeks of age in male chickens [15]. This could be explained by the fact that ME1 catalyzes the oxidative decarboxylation of malate and simultaneously generates reduced NAPD, which is involved in the de novo synthesis of fatty acid. According to our results, however, the expression of the *ME1* gene was mainly affected by the age at castration and not by androgen status, whereas the mRNA expression of the *PCK1* gene was mainly regulated by androgen status and not by age at castration. Therefore, we suggest that androgen status affects fat accumulation in male chickens by negatively regulating the expression of *PCK1* gene. Additionally, our microarray data found no differences in the expression levels of *LPL* and *HSL* genes between capons and intact male chickens, implying that avian species and mammals possess different mechanisms through which androgen status affects adipogenesis. This difference could be partly explained by the fact that the sites to lipogenesis are highly variable between avian species and mammals [13], [69]. For example, in mammals, the liver and adipose tissue are the 2 major sites of fatty acid production, whereas in avian species the liver is the main lipogenic site. Additionally, Human adipose tissue has a poor capacity to synthesize fatty acids de novo compared with that of the rat [70], which could explain the differences observed between humans and rats.

## Supporting Information

File S1 capon_vs_control_Result: upregulated and downregulated genes.
(RAR)

File S2 Affymetrix GeneChip technical manual.
(RAR)

File S3 all_expression_signal.
(RAR)
